# Health related quality of life assessment in acute coronary syndrome patients: the effectiveness of early phase I cardiac rehabilitation

**DOI:** 10.1186/s12955-016-0583-7

**Published:** 2017-01-13

**Authors:** Lawrence Anchah, Mohamed Azmi Hassali, Melissa Siaw Han Lim, Mohamed Izham Mohamed Ibrahim, Kui Hian Sim, Tiong Kiam Ong

**Affiliations:** 1 Department of Pharmacy, Sarawak Heart Centre, 94300 Kota Samarahan, Sarawak Malaysia; 2 Discipline of Social and Administrative Pharmacy, School of Pharmaceutical Sciences, Universiti Sains Malaysia, 11800 Penang, Malaysia; 3 Clinical Research Centre, Sarawak General Hospital, 94300 Kuching, Sarawak Malaysia; 4 Social and Administrative Pharmacy, College of Pharmacy, Qatar University, Al Tarfa, PO Box 2713, Doha, Qatar; 5 Department of Cardiology, Sarawak Heart Centre, 94300 Kota Samarahan, Sarawak Malaysia

**Keywords:** Clinical pharmacy, Cardiac rehabilitation programme, Quality of life, SF-36, Acute coronary syndrome

## Abstract

**Background:**

Acute Coronary Syndrome (ACS) is one of the most burdensome cardiovascular diseases in terms of the cost of interventions. The Cardiac Rehabilitation Programme (CRP) is well-established in improving clinical outcomes but the assessment of actual clinical improvement is challenging, especially when considering pharmaceutical care (PC) values in phase I CRP during admission and upon discharge from hospital and phase II outpatient interventions. This study explores the impact of pharmacists’ interventions in the early stages of CRP on humanistic outcomes and follow-up at a referral hospital in Malaysia.

**Methods:**

We recruited 112 patients who were newly diagnosed with ACS and treated at the referral hospital, Sarawak General Hospital, Malaysia. In the intervention group (modified CRP), all medication was reviewed by the clinical pharmacists, focusing on drug indication; understanding of secondary prevention therapy and adherence to treatment strategy. We compared the “pre-post” quality of life (QoL) of three groups (intervention, conventional and control) at baseline, 6 months and 12 months post-discharge with Malaysian norms. QoL data was obtained using a validated version of Short-Form 36 Questionnaire (SF-36). Analysis of variance (ANOVA) with repeated measure tests was used to compare the mean differences of scores over time.

**Results:**

A pre-post quasi-experimental non-equivalent group comparison design was applied to 112 patients who were followed up for one year. At baseline, the physical and mental health summaries reported poor outcomes in all three groups. However, these improved gradually but significantly over time. After the 6-month follow-up, the physical component summary reported in the modified CRP (MCRP) participants was higher, with a mean difference of 8.02 (*p* = 0.015) but worse in the mental component summary, with a mean difference of −4.13. At the 12-month follow-up, the MCRP participants performed better in their physical component (PCS) than those in the CCRP and control groups, with a mean difference of 11.46 (*p* = 0.008), 10.96 (*p* = 0.002) and 6.41 (*p* = 0.006) respectively. Comparing the changes over time for minimal important differences (MICD), the MCRP group showed better social functioning than the CCRP and control groups with mean differences of 20.53 (*p* = 0.03), 14.47 and 8.8, respectively. In role emotional subscales all three groups showed significant improvement in MCID with mean differences of 30.96 (*p* = 0.048), 31.58 (*p* = 0.022) and 37.04 (*p* < 0.001) respectively.

**Conclusion:**

Our results showed that pharmaceutical care intervention significantly improved HRQoL. The study also highlights the importance of early rehabilitation in the hospital setting. The MCRP group consistently showed better QoL, was more highly motivated and benefitted most from the CRP.

**Trial registration:**

Medical Research and Ethics Committee (MREC) Ministry of Health Malaysia, November 2007, NMRR-08-246-1401.

## Background

Acute coronary syndrome (ACS) is a type of cardiovascular disease that is generally used to describe a constellation of symptoms resulting in ischemic heart disease. The clinical spectrum of ACS ranges from the state of unstable angina (UA), non ST-elevation myocardial infarction (NSTEMI) to ST-elevation myocardial infarction (STEMI). A common presentation of patients diagnosed with UA and NSTEMI is typical ischaemic chest discomfort associated with transient non-ST-elevation echocardiography (ECG) changes. On the other hand, STEMI patients present with similar clinical symptoms, but of greater severity and are known to have ST-elevation on the ECG. This group of patients must undergo reperfusion intervention upon presentation.

Cardiac rehabilitation program (CRP) is primarily designed to counter the physiological and psychological burdens of heart disease. The main objectives of cardiac rehabilitation are to optimize patients’ physical functionality, to improve their quality of life, and to reduce recurrence of major cardiac and cerebrovascular events. Thus, CRP is driven to prolong and to improve quality of life (QoL), as reflected through improvements in physical functioning, well-being and in the alleviation of symptoms [1]. Less than half of all suitable patients are willing to participate in the aforementioned CRP. This has therefore highlighted that a newly proposed life-saving intervention should be coupled with evidence based secondary prevention to convince patients’ participation. Hence, encouraging all post ACS patients to attend this life-saving program.

Although we have compelling evidences on the benefits of cardiac rehabilitation, our referrals and attendance rates were very much lower than expected. Despite the documentation of significant morbidity and mortality benefits, cardiac rehabilitation activities were sadly underutilised and misjudged by many. Some patients may perceive that the cardiac rehabilitation as unnecessary or failing to meet their needs to recover or both. Moreover, those who were willing to participate at the initial stage tended to drop out from the programme earlier than scheduled. Special attention is needed for those at high risk of dropping out from the programme because early dropout from a CRP could be a compelling issue in psychologically-distressed patients, in patients of a younger age group and those who have poorer perceptions of their therapy plan.

Therefore, in order to improve the current CRP and to make it more interesting, an intensive evaluation involving innovative ideas from health care providers were of utmost importance. This hopefully will add impact to the alternative rehabilitation formats which in return is to increase awareness among post ACS patients. The success of CRP relies upon excellent services – from new innovative techniques to the application of scientific practical-based methods that aim for positive outcomes. These outcomes are typically measured from mobility or mortality reporting, attendance of the programme or from customer satisfaction surveys. From the patients’ perspective, psychosocial indices are related to cardiac symptoms and health-related quality of life (HRQoL) parameters. These are commonly used to measure the outcomes. Therefore, not only should we look at the performance indicators from the perspective of the healthcare system, we should also consider them from the patients’ perspective by measuring their HRQoL evaluation. HRQoL is an independent predictor of mortality and morbidity in patients who are suffering from post-acute coronary syndrome (ACS). A phase I cardiac rehabilitation programme, consisting of a multidisciplinary team approach that emphasises pharmacological, psychological and educational counselling, may be useful to improve patients’ understanding on their treatment plan and what to expect after discharging from a hospital. Hence, it is important that any information given is shared with the patients’ caregivers to avoid unnecessary confusion regarding their treatments and they can embark upon their journey to improve their quality of life.

The effectiveness of the early stages of phase I CRPs has not been extensively studied [2, 3]. For instance, interpretations of the findings from past studies have been poorly defined in terms of clinical significance, humanistic outcomes, and evaluation of cost effectiveness in the acute phase of patients with post-myocardial infarction. Thus, we aim to create an innovative CRP as an alternative programme that is comparable or may improve upon the existing conventional CRP. The evaluation of this programme will emphasize HRQoL issues and benefits to provide a better understanding of concomitant treatment post-ACS. In this modified programme (MCRP), associated clinical pharmacy services were introduced at the early stage of phase I, which is during the admission period. One of the services provided was a brief introduction on pharmaceutical care issues in the post-ACS phase. The efficacy of this specific clinical pharmacy intervention was measured using the HRQoL health outcomes scoring system. The roles of clinical pharmacists now involve a multi-disciplinary approach to cardiology care, improving knowledge of medication management, as well as enhancing the post-discharge care for post-ACS patients. To our knowledge, this is the first study in Malaysia to evaluate the efficacy of cardiology clinical pharmacy services in a phase I cardiac rehabilitation programmes. The findings from this study will benefit patients’ care in CVD management by reducing drug-related problems that may lead to unnecessary patients’ suffering, hence incurring huge costs to the society. For employers, improved patient care outcomes in HRQoL will lead to a decrease in days lost from work and thus increase productivity [4].

The aim of the study was to describe the effect and impact of early pharmaceutical care intervention in phase I and short course phase II cardiac rehabilitation on HRQoL.

## Methods

### Study design

Patients in the post ACS phase were enrolled from January 2008 to December 2010. The study protocol required completion of the SF-36 assessment, with a follow-up for 1 year, covering baseline, 6- and 12-month measurements. In order to detect the differences in outcomes between groups, a scale of 20 points difference is considered the minimal clinical important difference (MCID) [5, 6]. Therefore, a positive mean change against the baseline data at follow-up should be considered as an improvement in health and vice versa. This 20-point difference in this study generally is referred to our ACS patients.

### Data collection and assessment instruments

A questionnaire form, SF-36 version 1 was used to assess the quality of life score of post-ACS populations. This was done by comparing the relative burden of diseases in three treatment models. This trial determined patients’ views about their health over time. The questionnaire used is suitable for and comprehensible to lay persons in the Malaysian population, and has the additional advantage of being available in Malay version [7]. The SF-36 consists of eight separate domains: physical functioning (PF), role limitation because of physical health (RP), social functioning (SF), vitality (VT), bodily pain (BP), mental health (MH), role limitations because of emotional problems (RE) and general health (GH) with a standard scoring of 1 to 100 [8].

### Interventions and study participants

The recruitment of patients was based on a pre-post quasi-experimental non-equivalent groups’ comparison design. All the investigators and the clinicians were not blinded in this study. There were no restrictions to the clinicians in giving the treatment plan. He or she was free to recommend other specific interventions for ACS patients from other healthcare providers. Therefore, any medication counselling session, or reconciliation of patients on medication was recorded and noted down in patients’ folders by the clinical pharmacists. Patients’ understanding of their treatment needs is crucial and the role of pharmacists to disseminate the information is critical while patients are still in the ward. This intervention is done step-by-step according to the protocol to convey reassurance in adherence to treatment plan. Thus, the clinical pharmacists ensure that such medication counselling is covered from the basic idea of patients’ cardiac diseases to the understanding of drug-therapy and treatments. Those patients receiving intervention from pharmacists while in the ward were followed up until they had completed both phase I and Phase II of the CRP. The interview guide and the clinical pharmacists’ CRP manual were referred to as a standard checklist to make sure that all relevant information was collected and documented [9, 10]. During the initial stage, a survey was conducted via self-administration and interview for study participants in the hospital (e.g., in the Cardiac Care Unit, general ward, rehabilitation waiting area, examination room or pharmacy counselling room).

The inclusion criteria for ACS fulfilled at least the two conditions of clinical presentation with typical angina pain at rest and elevated cardiac biomarkers. We recruited those below 75 years of age who could participate in intensive exercise during the phase II CRP. Patients who had coronary angiography, where interventional treatment was indicated, or where coronary artery bypass grafting was planned, were invited to participate in the trial. The coronary angiography mentioned in this study covered primary, emergency or elective percutaneous coronary intervention. Those who presented with a severe co-existing medical condition, or residing at inaccessible areas were excluded from this study. Since most of the sessions in this programme contained exercise activities, our recruitment for the trial had to be selective. For those unable to do any exercise activities in this CRP were advised not to join in the full package of the cardiac rehabilitation programme. This exemption also applies to those who were categorised as high-risk patients whose treatment plans had yet to be resolved.

### Modified cardiac rehabilitation programme, Phase I

**Fig. 1:**
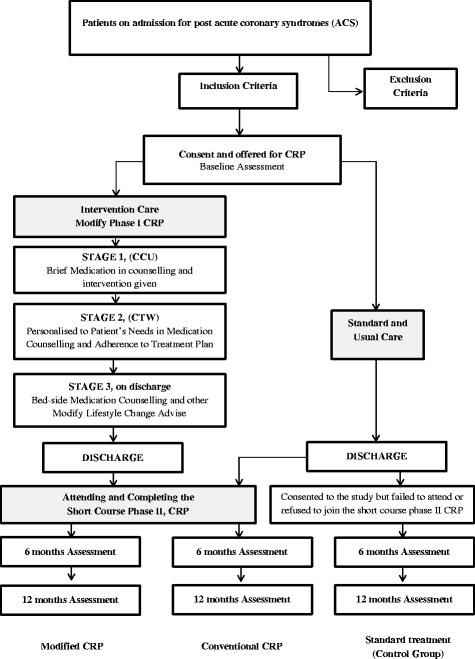
Recruitment protocol and selection for cardiac rehabilitation programmeᅟ

Phase I CRP is the inpatient programme that begins soon after a cardiac event (such as a heart attack, angioplasty, or a bypass surgery) and finishes when the patient is discharged. In modified CRP, clinical pharmacy services are added to the standard phase I CRP protocol. These services, emphasising on both education and medication adherence are mainly executed in all post ACS patients prior to their discharge. Therefore, intensive drug counselling sessions and pharmaceutical-care interventions are part of the services delivered by the clinical pharmacists. While patients are still in the ward, basic knowledge with regard to managing their angina is also part of the services that include lifestyle modifications and pharmacological treatments to reduce cardiovascular risks. In general, this process is called phase I modified CRP (or MCRP). Patients consenting to the study had their health assessed using a self-answered questionnaire. Phase I cardiac rehabilitation was delivered upon admission, involving a step-by-step counselling protocol delivered by a clinical pharmacist, and followed by the standard phase II cardiac rehabilitation programme, as MCRP [11]. However, due to a very short length of stay after admission, time limitation may be one of the many reasons that many could not be offered a phase I cardiac rehabilitation. Most of the ACS patients, however, were recruited to attend post-discharge cardiac rehabilitation. This is known as out-patient based phase II CRP (Fig. 1). We can therefore differentiate this group of patients from the other groups because the basic knowledge of their medication and treatment plan prior to discharge were recorded in every step of the counselling sessions provided by clinical pharmacists. In stage I of phase I CRP, a brief understanding of their disease with target therapy and information of each drug intervention made were delivered by a pharmacist. This is either done in an intensive care unit or cardiac care unit (CCU) once patients were stabilised and able to communicate with their healthcare providers. Once transferred to the general ward, information on their medication and treatment plan were once again delivered in depth by the clinical pharmacists, this time focusing more on personalising patients’ need in medication counselling and adherence to their treatment plan. Subsequently during the stage II phase I CRP, each pharmaceutical care issues previously addressed should be resolved prior to patients’ discharge. Stage III phase I CRP which covered all of their discharge medication until their next review or appointment involves the patients’ last contact with the pharmacist upon discharge. Therefore, another bed-side medication counselling were delivered to ensure and enforce medication therapy adherence, understanding the lifestyle modification changes and treatment plan which includes attending the outpatient CRP post-discharge. While in the ward, duration of contact with their patients was observed and recorded in their patients’ case notes. Pharmacists spent at least an hour with their patients each time they executed stage I, stage II and stage III of phase I CRP. All pharmaceutical care issues related to patients’ diseases and treatment had to be resolved by stage III phase I, otherwise the patient might have to stay in hospital for a while more until all prescribed treatment had been verified and endorsed.

### Phase II (Outpatient hospital-based)

This is an outpatient hospital-based programme. Patients normally begin their phase II CRP approximately 4 to 6 weeks after discharge from hospital. Phase II comprises multidisciplinary talks and exercise sessions that are based on the hospital schedule. This phase emphasizes education on disease, medication, diet, lifestyle modification and exercise intensity. The conventional publicly funded cardiac rehabilitation programme is a 4-week educational programme, scheduled for 2 days per week. All ACS patients were recommended to join in the phase II CRP. The educational session involves an interactive talk on relaxation, the risk factors for coronary heart disease as well as treatment and medication adherence. This educational session was conducted through group classes, slide presentations, and individual counselling. Individual education was provided by clinical pharmacists prior to physical exercise sessions and covered mainly pharmacotherapy, cardiovascular risk factors, and drug-related nutrition intake. On the other hand, those who were not willing to participate or who could not recruited into both phase I and phase II, were treated as the control group. Disqualification from recruitment could have been due to many reasons such as geographical or logistical issues, poor support from family, and others. Hence, patients who failed to meet the inclusion criteria for CRP were still consented and followed up without prejudice. These patients were treated as the usual care group [12–14]. All patients in the trial were given the standard therapy during admission and upon discharge. In this study, for the assessment section and follow up, all the three groups were consented and assessed at baseline, 6 and 12 months and treated with standard therapy according to good clinical practice guidelines (Fig. 1).

### Statistical analyses

The norm-based interpretation method was used to interpret the SF-36 scale score, whereby interpretation is based on defining the differences between the mean of the based-norm score and the mean of group score. One-way analysis of variance (ANOVA) was used to verify the groups’ homogeneity. The mean differences between baseline, 6-month, and 12-month measurements were analysed for each of the three groups. These intervals were used in most of the domains. We considered that some domains may have changed remarkably within a few months. However, follow-up data after a year and assessments of each patient were also considered a completion of quality of life analysis for comparison for each group and within the groups themselves. It was essential to do the annual assessment of current clinical progress together with quality of life assessments. Thus, the differences between baseline, 6-month, and 12-month measurements were essential. Post-hoc multiple comparison analysis was used to identify the differences between the three groups at the same intervals. A Kruskal-Wallis test was used to evaluate the differences in non-parametric data between the groups.

The score across all eight domains along with the two scores for the physical and mental summaries were used as a general linear model of repeated measurement for the three different time frames for the treatment groups. The sphericity assumption was applied in SPSS 16.0 software for Windows and a probability value of *p* < 0.05 was considered as statistically significant.

## Results

### Patient characteristics

A total of 112 patients met the eligibility criteria for the study (Fig. 1). Sixty-two patients recruited were categorised as non-rehabilitation participants while 50 patients (44.6%) were recruited for phase II CRP. All patients were divided into three groups. Twenty-two patients were recruited for the modified model of phase I CRP and subsequently underwent a short course of phase II CRP upon discharge. In the conservative CRP group, 28 patients were recruited to participate only in the outpatient short course phase II CRP, while sixty-two patients were in the usual care or control group (Table 1).

**Table 1:** Baseline characteristics of 112 patients in three groups

	MCRP (*n* = 22)	CCRP (*n* = 28)	Control (*n* = 62)	*p* value
Demographic characteristics				
Gender (Male: Female)	21:1	24:4	54:8	0.259^a^
Age in years, mean (SD)	52.73 (10.47)	57.92 (9.21)	56.85 (10.71)	
Length of stay, no. (%)				
More than median of 4 days	12 (55)	10 (36)	19 (29)	-
Educational level, no. (%)				
1. Primary/No formal education	9 (40.8)	18 (64.3)	37 (61.6)	0.454^b^
2. Secondary school or college	12 (57.1)	10 (35.7)	23 (38.4)	
Physical characteristics, mean (SD)				
Body weight (kg)	66.20 (12.49)	67.15 (8.96)	67.85 (12.81)	0.847^a^
BMI (kg.m ^−2^)	25.08 (4.22)	26.08 (3.32)	26.11 (3.83)	0.417^a^
Waist & hip ratio	0.92 (0.03)	0.92 (0.16)	0.96 (0.13)	0.267^a^
Systolic on admission	133.09 (27.53)	142.04 (30.18)	142.35 (29.63)	0.556^a^
Diastolic on admission	81.85 (17.29)	81.20 (17.32)	83.31 (18.32)	0.994^a^
Heart Rate on admission	85.52 (31.96)	79.44 (17.90)	83.07 (22.66)	0.889^a^
Ejection fraction	50.06 (10.71)	50.81(12.45)	49.59 (12.72)	0.932^a^
ACS Stratum, no. (%)				
STEMI	11 (50.0)	17 (60.7)	33 (53.2)	0.735^b^
NSTEMI	8 (36.3)	3 (10.7)	14 (22.5)	0.104^b^
UA	3 (13.6)	9 (32.1)	20 (32.2)	0.232^b^
Current smoker, no. (%)	11 (52.5)	9 (32.2)	21 (35)	0.422
Fagerström test mean (SD)	**5.45** (2.29)	4.67 (2.87)	4.7 (3.04)^c^	*p* < 0.05
Family support, no. (%)	19 (95)	26 (92.8)	57 (98.2)	0.443

Continuous values are expressed as mean (SD) or number (percentage). One-way analysis of variance (ANOVA) was used. *BMI* Body Mass Index, *STEMI* ST elevation MI, *NSTEMI* Non-ST elevation MI and *UA* Unstable angina, *MCRP* Modified CRP, *CCRP* Conventional CRP

^a^Kruskal–Wallis

^b^χ^2^-test for nominal data (frequencies)

^c^Statistical significance was fixed at *p* < 0.05

The clinical examination and physical characteristics were similar across all the three groups. Antrometry measurements of body mass index (BMI) were 25.90 ± 3.75 kg/height (m)^2^ (range, 18.13 to 36.36) indicating a slightly higher than the ideal BMI score of 25. Most of the patients in this trial also demonstrated a high waist to hip ratio 0.95 ± 0.05 (range, 0.83 to 1.12) at baseline [15]. During the management of the acute stage, those presenting with high blood pressure may pose challenges to clinicians and clinical pharmacists. In this cohort, we observed that the mean systolic BP (140.33 ± 29.29), and diastolic BP (82.48 ± 17.70) were slightly higher than normal.

### Population norms comparison as anchor-based methods to determine changes

**Fig. 2:**
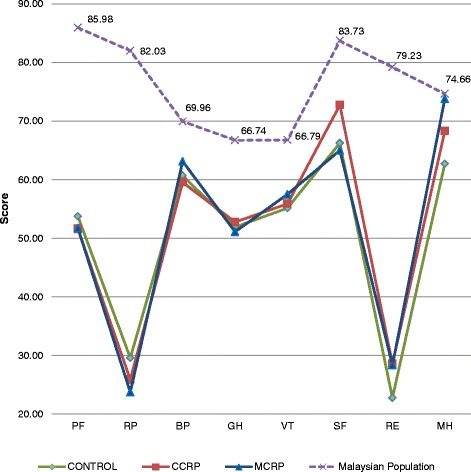
Distribution of eight domain scores at baseline assessment between three groups and the Malaysian population norms

Eight subscales of health status data were collected and compared with the Malaysian general population norms [16]. At baseline, they scored below the mean matched population values on all domains. In general, patients post-ACS exhibited significantly lower QoL scores compared to the population norms (Fig. 2). All three groups showed impairment in physical functioning (mean differences −32.2 in the control group, −34.3 in the CCRP group, and −34.2 in the MCRP groups). For role physical, mean differences were −52.4, −56.1 and −58.2, respectively, and, for role emotion domains, mean differences were −56.4, −50.6 and −50.9, respectively. However, in the control group, the role emotion domain mean change deficit was statistically significant (mean differences −56.4, *p* = 0.032) compared with the CCRP (−50.6) and MCRP (−50.9) points. Smaller, but significant, mean differences were noted for bodily pain (mean difference −9.26 in control group, −10.4 in the CCRP group and −6.81 in the MCRP groups), general health perception (mean differences were −14.8, −13.9 and −15.6, respectively), energy and vitality (mean differences were −11.6, −10.9 and −9.29, respectively), and social functioning (mean differences were −17.4, −10.9 and −18.7, respectively) (Table 2).

**Table 2:** Quality-of-life scores obtained by comparing with normative values after the 12 months follow-up assessment

Differences between means for the three arms and Malaysian population norms^a^							
Normative value^b^		MCRP		CCRP		Control	
Domains		Mean	95% CI	Mean	95% CI	Mean	95% CI
PF	85.98 ± 17.91	*−2.98* ^*c*^	−13.64 to 7.68	−3.87	−13.11 to 5.36	−16.0	−27.09 to −4.90
RP	82.03 ± 32.12	*−20.36* ^*c*^	−42.46 to 1.75	−22.81	−43.38 to −2.25	−30.3	−46.36 to-14.24
BP	69.96 ± 17.59	*10.57* ^*c,d*^	−2.09 to 23.23	3.72^d^	−8.94 to 16.39	−4.16	−14.92 to 6.59
GH	66.74 ± 19.99	*3.66* ^*c,d*^	−9.88 to 17.20	1.10^d^	−10.34 to12.54	−2.87	−12.96 to 7.21
VT	66.79 ± 17.68	*6.21* ^*c,d*^	−6.16 to 18.58	1.36^d^	−941 to 12.15	−1.96	−12.67 to 8.75
SF	83.73 ± 19.23	−2.89	−13.95 to 8.16	*−1.49* ^*c*^	−12.91 to 9.92	−15.62	−27.65 to −3.59
RE	79.23 ± 35.92	*−17.01* ^*c*^	−42.03 to 8.01	−23.08	−43.16 to −3.01	−33.25	−49.29 to-17.21
MH	74.66 ± 17.19	*9.34* ^*c, d*^	1.13 to 17.54	7.23^d^	0.60 to 13.86	−0.59	−9.17 to 7.99

The differences between means for patient groups tested at 12 months follow-up and the population norms

^a^Calculated from the differences of two respective means (mean of group minus mean of normative value)

^b^Value derived from a sample of 3072 healthy participants studied by Azman et al. Malaysian population norms as a reference point

^c^Mean difference is higher in value which indicates a better health score between the three groups

^d^Positive value of mean differences was considered higher than the normative value

### Population norms comparison at 6-month follow-up

In this analysis means differences were used to compare and to describe the descriptive data of each domain over time. The role physical and role emotion domains did not improve markedly at the 6-month assessment (mean differences −40.1 in the control group, −38.9 in the CCRP group and −36.7 in the MCRP group for the role physical domain; and −34.1, −31.0 and −39.6, respectively, for the role emotion domain). Overall, at the 6-month assessment, most of the eight domains in post-ACS patients’ QoL were still alarmingly poor. The negative means against the normative data reflects a decline in scores and deterioration in health. Thus, within the 6-month period, after a long follow-up, medication intake and even, for some, cardiac rehabilitation intervention, the recovery period and the healing process after an acute event of ACS had not yet reach the point of full recovery.

### Comparison at 12-month follow-up

At the 12 month follow-up, overall domains were still below the population norms. The cardiac rehabilitation groups performed better than the control group: in the physical functioning domain the mean differences (−3.87 for CCRP and −2.98 for MCRP groups), were better compared to the control group (mean difference −14.1). In addition, the social functioning mean differences for the CCRP (−1.49) and MCRP groups (−2.9), were also better compared to the control group (mean difference −13.8). At the 12-month follow-up the QoL had improved, especially in respect to bodily pain, general health, vitality and mental health subscales. These positive mean differences should be interpreted as major health improvements.

In the MCRP and CCRP groups, four domains with positive mean differences were observed (bodily pain, general health, vitality and mental health) with higher values in the MCRP group (mean differences 10.57, 3.66, 6.21 and 9.34, respectively) compared to the CCRP group (mean differences 3.72, 1.10, 1.37 and 7.23, respectively).

### Comparison of baseline and 6-month MCID

#### MCRP group

Fifteen patients (68%) were analysed for paired *t*-test and MCID evaluation [17–19]. During this initial 6 month period, the MCRP group demonstrated very low values in the mental health domain (mean difference −10.67), and therefore contributed a low score to the mental component summary (MCS) (mean difference −4.13, 95% CI, −10.28 to 2.03). The other seven domains in the MCRP group, however, did show improvements. The MCRP group showed a relatively higher score in physical functioning (mean difference 17.22), while the role physical domain had the highest score with a statistically significant mean difference of 25 points (*p* = 0.03). The percentages of mean difference for the physical functioning and role physical domains were 34.21% and 149.97% respectively. Both domains therefore contributed to an improvement in the physical component summary (PCS) score with a statistically significant mean difference of 8.02 point (*p* = 0.015) (Table 3).

**Table 3:** Comparison of mean changes and percentage changes between baseline and 6 months’ follow-up from the three groups

	MCRP (*n* = 15)		CCRP (*n* = 18)		Control (*n* = 33)	
	Mean difference	(%)	Mean difference	(%)	Mean difference	(%)
Components						
PCS	8.02	*22.16* ^*a,b*^	5.30	13.86	2.72	6.70
MCS	−4.13	−9.03	1.84	3.86	2.80	*6.62* ^*a*^
Domains						
PF	17.22	34.21	*22.78*	*45.56* ^*a,b*^	11.97	20.36
RP	*25.00* ^*c*^	*149.97* ^*a,b*^	12.50	40.90	18.18	*72.73* ^*b*^
BP	3.20	4.92	12.67	*22.18* ^*a*^	4.67	7.30
GH	7.09	*14.14* ^*a*^	2.61	4.78	−0.09	−0.16
VT	1.00	*1.76* ^*a*^	1.11	1.69	−0.25	−0.44
SF	5.00	*8.11* ^*a*^	0.00	0.00	3.41	5.06
RE	6.68	23.14	18.53	62.53	*27.27* ^*c*^	*142.12* ^*a,b*^
MH	−10.67	−14.76	10.44	*15.51* ^*a*^	3.27	4.98

^a^Highest percentage of mean differences among the three groups

^b^ *p* value for paired *t*-test is significant at *p* < 0.05

^c^MCID with reference point as 20-points differences in changes over time with the SF-36 (Ware et al., 1993; Wyrwich et al., 2004; Kemmler et al., 2010)

#### CCRP group

Eighteen patients (64.2%) were analysed. The CCRP group showed a statistically significant mean difference for physical functioning of 22.78 (95% CI, 4.82 to 40.73) which exceeded the MCID score. Thus, at the 6-month assessment, both CCRP and MCRP physical functioning and role physical domains had improved, signifying that CRP can help patients’ recover physical capacity significantly.

#### Control group

Only thirty-three patients (53.2%) were analysed for the 6 month assessment, and only the role physical (mean difference 18.18), and role emotional (mean difference 27.27) achieved the MCID. These findings suggest that those in the non-CRP group had a poorer perception of disease, indicating that relevant information may not have been conveyed well to this group.

### Comparing the mean difference of the three groups over time

**Fig. 3:**
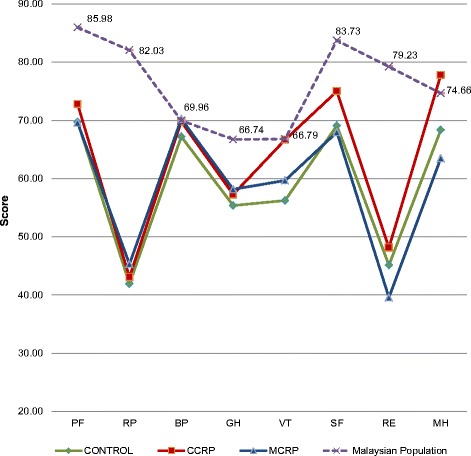
Distribution of eight domain scores at 6 months’ assessment between three groups and the Malaysian population norms

The physical component summary reported in the MCRP participants was higher among the three groups, with a mean difference of 8.02 (22.16%) (*p* = 0.015). Higher percentage values were noted in the MCRP group for role physical, general health, vitality, and social functioning (149.97%, 14.14%, 1.76%, and 8.11%, respectively). In the CCRP group, participants performed better on physical functioning, bodily pain and mental health (PF = 45.56%, B*p* = 22.18%, and MH = 15.51%, respectively). In the control group, however, only role physical (R*p* = 72.73%) and role-emotional (RE = 142.12%) were reported to show better results (Table 3). These findings showed that MCRP participants reported better HRQoL at the 6-month assessment (Fig. 3).

### Comparisons of baseline and 12-month MCID

#### MCRP group

HRQoL was determined for the two-time period comparisons at baseline and 12 month follow-up. Fourteen patients (63.6%) had improvements in their PCS, with a mean difference of 11.46 (95% CI, 3.46 to 18.85, *p* = 0.008). This was largely contributed by the three domains in physical health status: physical functioning (mean difference 30.36, 95% CI, 13.62 to 47.09, *p* = 0.002), physical role functioning (mean difference 41.07, 95% CI, 11.32 to 70.81, *p* = 0.011), and general health (mean difference 16.28, 95% CI, 2.28 to 31.57, *p* = 0.027). In MCRP, the mentality subscale showed a higher vitality domain (mean difference 14.64, 95% CI, 0.12 to 29.16, *p* = 0.048), social functioning (mean difference 20.53, 95% CI, 2.27 to 38.79, *p* = 0.03) and mental health (mean difference 11.43, 95% CI, 0.11 to 22.73, *p* = 0.048); along with reported MCID in social functioning and role-emotional subscales (30.96, 95% CI, −0.58 to 62.51).

#### CCRP group

More than half of the participants (*n* = 19, 71.5%) were analysed for their progression from baseline until the 12-month follow-up. Their PCS scores (mean difference 10.96, 95% CI, 4.6 to 17.32, *p* = 0.002) improved largely due to the contributions by these four subscales: physical functioning (35.79, 95% CI, 20.91 to 50.67, *p* < 0.001), role physical (32.89, 95% CI, 6.85 to 58.94, *p* = 0.016), bodily pain (20.53, 95% CI, 4.92 to 36.13, *p* = 0.013) and general health (18.0, 95% CI, 4.5 to 31.50, *p* = 0.012). We also found that patients in CCRP group had reported MCS scores (mean difference 6.71, 95% CI, 1.17 to 12.24, *p* = 0.02) that were significantly better than baseline. This recovery in mental status was due to role emotional (31.58, 95% CI, 5.09 to 58.08, *p* = 0.022) and mental health outcomes (19.37, 95% CI, 6.82 to 31.92, *p* = 0.005). Overall, the improvement of QoL in PCS and MCS was reflected from four domains (PF, RP, BP and RE) which also achieved MCID.

#### Control group

Only 27 patients (43.5%) completed the questionnaires for analysis. The physical status achievement was found to have improved due to physical functioning (20.16, 95% CI, 6.41 to 33.91, *p* = 0.006), role physical (36.11, 95% CI, 17.85 to 54.37, *p* < 0.001) and general health (13.26, 95% CI, 1.31 to 25.21, *p* = 0.031), while the mental status achievement improved due to vitality (13.52, 95% CI, 3.5 to 23.54, *p* = 0.01), role emotional (37.04, 95% CI, 18.27 to 55.81, *p* < 0.001) and mental health (17.48, 95% CI, 5.83 to 29.14, *p* =0.005) (Table 4).

**Table 4:** Comparison of mean changes and percentage changes between baseline and 12 months’ follow-up for the three groups

	MCRP (*n* = 14)		CCRP (*n* = 19)		Control (*n* = 27)	
	Mean difference	(%)	Mean difference	(%)	Mean difference	(%)
Components						
PCS	11.46	*31.44* ^*a,b*^	10.96	*30.25* ^*b*^	6.27	*16.08* ^*b*^
MCS	5.9	13.09	6.71	*15.35* ^*b*^	7.96	*19.84* ^*a,b*^
Domains						
PF	*30.36* ^*c*^	*59.03* ^*b*^	35.79^c^	*77.27* ^*a,b*^	*20.16* ^*c*^	*37.55* ^*b*^
RP	*41.07* ^*c*^	*229.96* ^*a,b*^	32.89^c^	*124.98* ^*b*^	*36.11* ^*c*^	*185.76* ^*b*^
BP	16.57	26.48	20.53^c^	*38.61* ^*a,b*^	9.96	16.73
GH	16.93	32.83^b^	18.00	*36.12* ^*a,b*^	13.26	*24.54* ^*b*^
VT	14.64	*25.95* ^*a,b*^	12.11	21.60	13.52	*24.83* ^*b*^
SF	*20.53* ^*c*^	*34.84* ^*a,b*^	14.47	21.36	8.80	13.87
RE	*30.96* ^*c*^	108.42	31.58^c^	*128.60* ^*b*^	*37.04* ^*c*^	*300.17* ^*a,b*^
MH	11.43	*15.94* ^*b*^	19.37	*30.97* ^*a,b*^	17.48	*29.28* ^*b*^

^a^Highest percentage of mean differences among the three groups

^b^ *p* value for paired *t*-test is significant at *p* < 0.05

^c^MCID with reference point as 20-points differences in changes over time with the SF-36 (Ware et al., 1993; Wyrwich et al., 2004; Kemmler et al., 2010)

### General QoL findings between the three groups

**Fig. 4:**
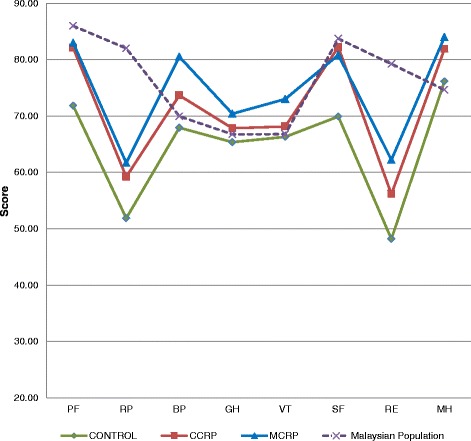
Distribution of eight domain scores at 12 months’ assessment between three groups and the Malaysian population norms

The highest percentage values of mean differences among the three groups were noted in both of the cardiac rehabilitation groups (Fig. 4). The MCRP group contributed the highest percentage mean differences in the physical, role physical, vitality and social functioning components (PCS = 31.44%, R*p* = 229.96%, VT = 25.95% and SF = 34.84%), followed by CCRP in physical functioning, bodily pain, general health, and mental health (PF = 77.27%, B*p* = 38.61%, GH = 36.12% and MH = 30.97%) (Table 4).

## Discussion

### Physical Health Outcomes

Studies on post-ACS patients with gradual but intensive increases in physical fitness during CRP have shown that physical, psychological and social recovery become increasingly obvious and statistically significant, from 3 to 6 months. This indicates that 6 months of exercise training in CRP induces significant improvements in ventricular remodelling and autonomic tone in patients with acute myocardial infarction and percutaneous coronary intervention. Furthermore, improvements in physical fitness correlated well with positive psychometric scores, improvements in exercise-capacity and QoL [20, 21]. Ades et al. (2006) stresses the importance of cardiac rehabilitation on high level improvements of initial physical disability found among post-ACS participants in CRP [22, 23]. This implies that patients without CRP with known low levels of baseline fitness score can lead to a very poor prognoses. Most of the domains in the control group were statistically significant following the 12-month assessment period as demonstrated in their physical activities scores inclusive of role physical, general health and vitality. On the other hand those domains were not superior to the scores achieved by the rehabilitation participants. The paired *t*-test analysis at baseline and at 6 months showed that MCRP participants reported a very high score on the role physical subscale with a statistically significant mean difference of 25 points (*p* = 0.03), which remained high until the final assessment at the twelfth month follow-up. Overall, physical activities in MCRP patients were markedly improved at the sixth and twelfth month follow-up (Table 5).

**Table 5:** Comparison of mean changes and percentage changes between 6 months’ and 12 months’ follow-up for the three groups

	MCRP (*n* = 15)		CCRP (*n* = 15)		Control (*n* = 26)	
	Mean difference	(%)	Mean difference	(%)	Mean difference	(%)
Components						
PCS	3.95	8.90	4.26	*9.69* ^*a*^	3.69	*9.12* ^*b*^
MCS	9.23	*21.75* ^*a,b*^	3.32	6.89	4.69	10.90
Domains						
PF	14.78	*21.67* ^*a*^	7.67	10.27	6.32	9.76
RP	16.67	37.04	16.67	40.00	15.38	*42.10* ^*b*^
BP	12.27	*17.97* ^*a*^	4.60	6.56	6.38	10.20
GH	12.51	*21.62* ^*b*^	13.87	*25.36* ^*b*^	14.58	*28.52* ^*a,b*^
VT	14.33	24.43	8.00	12.63	13.97	*26.58* ^*a,b*^
SF	14.17	*21.25* ^*a*^	6.67	8.79	5.77	9.09
RE	*22.22* ^*c*^	*55.55* ^*a*^	8.88	19.03	12.83	34.50
MH	*21.07* ^*c*^	*33.48* ^*a,b*^	6.93	*9.19* ^*b*^	9.23	13.86

^a^Highest percentage of mean differences among the three groups

^b^ *p* value for paired *t*-test is significant at *p* < 0.05

^c^MCID with reference point as 20-points differences in changes over time with the SF-36 (Ware et al., 1993; Wyrwich et al., 2004; Kemmler et al., 2010)

At the 12-month follow-up it was obvious that rehabilitation participants had better outcomes with higher scores in the physical domains [24]. The mean difference of the physical component score (PCS) in MCRP was statistically significant (*p* = 0.015) at baseline and 6 months, and remained so at the final 12-month assessment (*p* = 0.008). The educational and motivational interview conducted while patients were hospitalised, along with some extra motivation entities, significantly improved and optimised the functional gains in the MCRP group. While patients were still hospitalised, the in-patient counselling on medication plan and time spent with patients to discuss their CAD treatment were seen as important activities and this shows that intervention and counselling by pharmacists were able to positively influence patients’ participation in CRP [25–27].

The policy of discharging AMI patients earlier (in fewer than 4 days) without any interdisciplinary intervention by health care providers while they are on admission is claimed to be cost-saving. However, in the long run, the QoL of these patients will be compromised, leading to its own cost implications [28, 29]. In this study, the majority of the patients (*n* = 12, 55%) in the MCRP group remained in hospital for longer (median > 4 days) but such prolonged stays in intensive care wards must not be taken per se as a negative indicator and does not necessarily lead to future poorer HRQoL [30]. On the other hand, longer stays may allow more time for clinical pharmacists to practise pharmaceutical care interventions, medication counselling and therapeutic agents optimisation which have been associated with improved long-term prognoses [31, 32]. This study shows the benefits of phase I CRP in bridging the gap in the provision of educational activities to ACS patients.

### Mental health outcomes

Psychological distress is another predicament which is very difficult to treat and resolve in MI patients. In this study, the non-accessibility to rehabilitative resources coupled with suboptimal social and family support have been shown to be stress factors affecting many patients in CRP leading to poor medical outcomes [33, 34].

Psychological adjustment may take up to 1 year for improvements to become evident [35]. The improvements in mental health subscale were observed for MCRP patients at the 12- month assessment in this study and were statistically significant (mean difference 21.07, *p* = 0.007). Overall, the mental component (MCS) score reported in the MCRP patient group showed the highest improvement among the three groups. CCRP participants also performed much better compared to the control group, but less well than the MRCP patients. Yonezawa et al. (2009) studied post MI patients following cardiac rehabilitation and found that after more than 6 months of follow up they rectified job stress and HRQoL slowly picked up [36]. Improvements in the mental health component subscale for both categories of cardiac rehabilitation participants (MCRP and CCRP) proved that cardiac rehabilitation intervention is clinically useful in reducing psychological stress and anxiety among ACS patients, as well as enhancing QoL scores [37, 38].

Overall, pharmacological, psychological and educational interventions in early phase I (inpatient) significantly reduce patient anxiety upon discharge. The substantial improvement of QoL, especially in the MCS score component reported in the MCRP group compared to the CCRP group further supports the positive contribution of clinical pharmacy work in phase I hospital-based CRP [39].

In summary, without cardiac rehabilitation intervention, QoL in ACS patients did not reach a satisfactory level and remained below the population norms during the study period (Table 2). Social functioning and role limitation due to emotional problems also remained poor in those without cardiac rehabilitation and the scores were worse than other population norms found elsewhere [40, 41].

### Limitations

The present study has several limitations: the rehabilitation allocation was based on the patients’ vicinity and accessibility to the centre. The MCRP group had a high attendance rate and was well-motivated, and this could have influenced the positive results in this study. ACS patients were selected from only one referral hospital in east Malaysia and therefore may not be generalizable to the entire population of the country.

## Conclusions

The provision clinical pharmacy services in the early stage of in-patient hospital-based cardiac rehabilitation has shown a practical improvement in physical activities and mental health at 1 year follow-up in post-ACS patients. The QoL after cardiac rehabilitation intervention seemed to be superior to the population norms in terms of physical fitness and enhancement of positive mental health function.

The MCRP confirmed the humanistic long-term benefits of phase I hospital-based and phase II outpatient cardiac rehabilitation. This model of activities can be a catalyst for further development of specialist clinical pharmacy interventions in cardiology and therefore may have fundamental value for the health care system in Malaysia.

## Abbreviations

ACS: Acute Coronary Syndrome
ANOVA: One-way analysis of variance
BP: Bodily pain
CCRP: Conventional Cardiac Rehabilitation Programmes
CRP: Cardiac Rehabilitation Programmes
GH: General health
HRQoL: Health-related quality of life
MCID: Minimal clinical important difference
MCRP: Modified Cardiac Rehabilitation Programmes
MCS: Mental component summary
MH: Mental health
PCS: Physical component summary
PF: Physical functioning
QoL: Quality of life
RE: Role limitations because of emotional problems
RP: Role limitation because of physical health
SF: Social functioning
SF-36: Short-Form 36 Questionnaire
VT: Vitality
